# Study of Highly Efficient Au/Pt Nanoparticles for Rapid Screening of *Clostridium difficile*

**DOI:** 10.1021/acsomega.4c00722

**Published:** 2024-05-24

**Authors:** Ying-Tsang Lu, Yu-Xlang Zeng, Wu-Xiong Tsai, Hsin-Chang Huang, Ming-Yuan Tsai, Yong Diao, Wei-Hsuan Hung

**Affiliations:** † School of Medicine, Huaqiao University, No. 269 Chenghua North Rd ,Quanzhou ,Fujian 362021, China; ‡ Institute of Material Science and Engineering, National Central University, No. 300, Zhong-da Rd. Zhongli District ,Taoyuan City 32001, Taiwan (R.O.C.); § Strong Biotech Corporation, 7f., No. 32, Sec. 1, Chenggong Rd., Nangang District ,Taipei City 11570, Taiwan (R.O.C.); ∥ Tripod Nano Technology Corporation, No. 3, Gongye 12th Rd., Pingzhen District ,Taoyuan City 324403, Taiwan (R.O.C.)

## Abstract

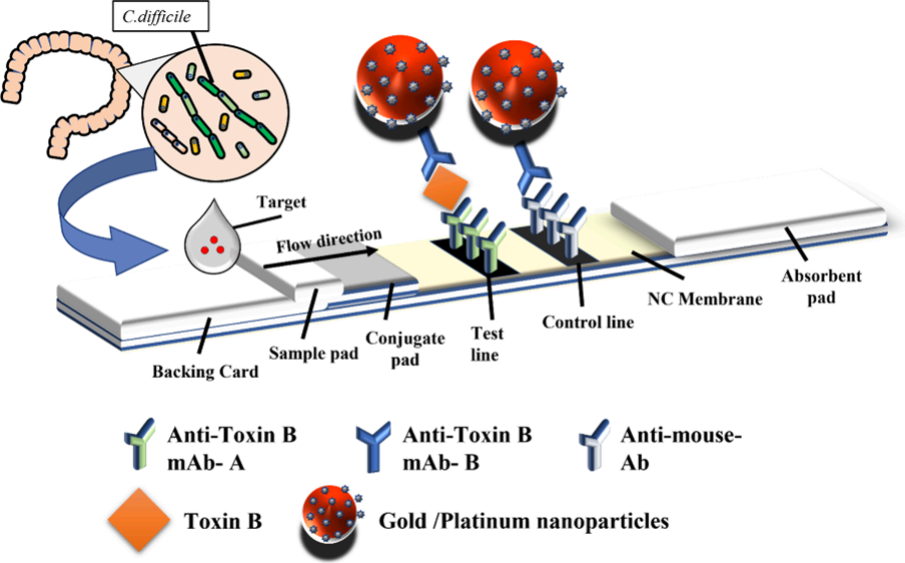

This study synthesized core/shell gold–platinum nanoparticles and characterized their colorimetric properties; ultraviolet-visible spectroscopy revealed that the synthesized nanoparticles exhibited distinct colors from conventional gold nanoparticles. Furthermore, the nanoparticles were subjected to lateral flow assays using Protein A, and the results revealed that they outperformed conventional spherical gold nanoparticles in terms of color development. This improvement can be attributed to the distinct core/shell structures of our nanoparticles. Further evaluation revealed that these nanoparticles could facilitate the detection of *Clostridium difficile* Toxin B visually at an extremely low concentration (1 ng/mL) without the requirement for advanced instrumentation. This substantial improvement in sensitivity can be attributed to the meticulous design and nanoscale engineering of the structure of the nanoparticles.

## Introduction

Gold nanoparticles have seen widespread application across various fields owing to their remarkable properties.^1,2^ Gold is inherently stable and thus exhibits minimal reactivity with other substances. Additionally, it exhibits excellent biocompatibility,^3,4^ making it safe for use within the human body without triggering rejection reactions. This has thus catalyzed advancements in the biological applications of gold nanoparticles. Gold nanoparticles possess unique optical characteristics^5,6^ due to surface plasmon resonance, imparting a range of captivating colors not seen at larger scales.

These properties render gold nanoparticles invaluable in diagnostic applications, particularly as carriers and signal sources in rapid screening reagents. Their red signals complement those of the human body,^7^ facilitating the achievement of effective detection processes. However, in lateral flow assays (LFAs) used for rapid screening, signals emitted by gold nanoparticles are weak, limiting the interpretability of the results. Increasing the size of such nanoparticles could enhance visibility, but it could hinder their flow on the test strip.^8,9^ Nevertheless, gold nanoparticles are appreciated for their low interference, which reduces the necessity for amplifiers. The advancement of LFAs faces challenges because of the need for improved detection methods in the face of epidemics, such as drug-resistant bacterial strains and COVID-19. These challenges underscore the urgent need for innovative rapid screening techniques.

Recently, the core/shell structured nanomaterials have become one of the most popular research subjects owing to their exceptional physicochemical properties and multifunctional composition, which can be used for many applications such as in biomedicine, pharmaceuticals, biosensing, optics, electronics, and catalysis.

Mixed noble metal nanoparticles, composed of gold, platinum, and silver, exhibit remarkable stability even under harsh conditions. As a result, they hold great promise as materials for enhancing signals in colorimetric assays.^10^ Platinum-modified gold (Au/Pt) nanoparticles have the original properties of AuNP and catalytic properties of a platinum layer. Hence, it is even more convenient in *in vitro* diagnostics, especially in catalytic activity and color performance.^11−14^

*Clostridium difficile* is a bacterium endemic to the human gut and can spread through the air. The incidence of *C. difficile* infections has increased in recent years. The symptoms of such infections vary depending on the patient’s health status and can include diarrhea, stomach pain, and even digestive tract perforation. To prevent the rapid spread of such infections, various biomedical assays have been developed. These include polymerase chain reaction (PCR) assays,^15^ the enzyme-linked immunosorbent assay,^16^ and LFAs.^17,18^ Although PCR assays provide accurate quantitative and qualitative analyses, they depend on specialized personnel and precision instruments that are often scarce in resource-limited regions. Consequently, LFAs are favored in such regions owing to their convenience, speed, low cost, and user-friendliness.

Since 2020, with the rapid spread of COVID-19, the demand for engineering nanoparticle applications in LFAs has increased.^19^ Research is primarily directed toward refining the colorimetric potential of nanoparticles by modifying their surfaces and shapes. The aim of this modification is to increase the adsorption of detection proteins on the nanoparticle surface, enhance detection sensitivity, reduce the nanoparticle quantities required, and ultimately lower costs while improving the assay sensitivity.

## Experimental Methods

### Experimental Design

In this study, we developed novel nanoparticle composite materials. We modified the surface of the gold nanoparticles to change the light absorption range and intensity, producing colors distinct from those of traditional colloidal gold. By coating gold nanoparticles with spherical platinum nanoparticles, we increased the surface area of the nanoparticles, thereby improving the LFA sensitivity. This modification can reduce raw material consumption, resulting in more economical detection materials for LFAs.

### Synthesis Method

#### Synthesis of Gold Nanoparticles

We employed the Turkevich method^20^ to synthesize gold nanoparticles. We stirred 150 mL of 2.2 mM sodium citrate dihydrate solution with a magnetic heating stirrer, while the mixture was brought to a boil. Next, we added 1 mL of a 25 mM chloroauric acid solution to the boiling mixture and allowed the reaction to proceed for 30 min to form gold nanocrystals. To enable the growth of the gold nanoparticles, we reduced the temperature to 90 °C, added 1 mL of 60 mM citric acid dihydrate, and then added 1 mL of 25 mM chloroauric acid consecutively. We repeated these steps to obtain successive generations (denoted as G3, G4, and G5) of gold nanoparticles.

#### Synthesis of Gold–Platinum Nanoparticles

We stirred the previously synthesized gold nanoparticles (G3, G4, and G5) with a magnetic heating stirrer at a high temperature. We then added 2 mL of 56.8 mM ascorbic acid solution followed by 1 mL of 25 mM chloroplatinic acid solution, thus forming G3Pt, G4Pt, and G5Pt gold–platinum nanoparticles.^21^

#### Conjugation Process

**Figure 1:**
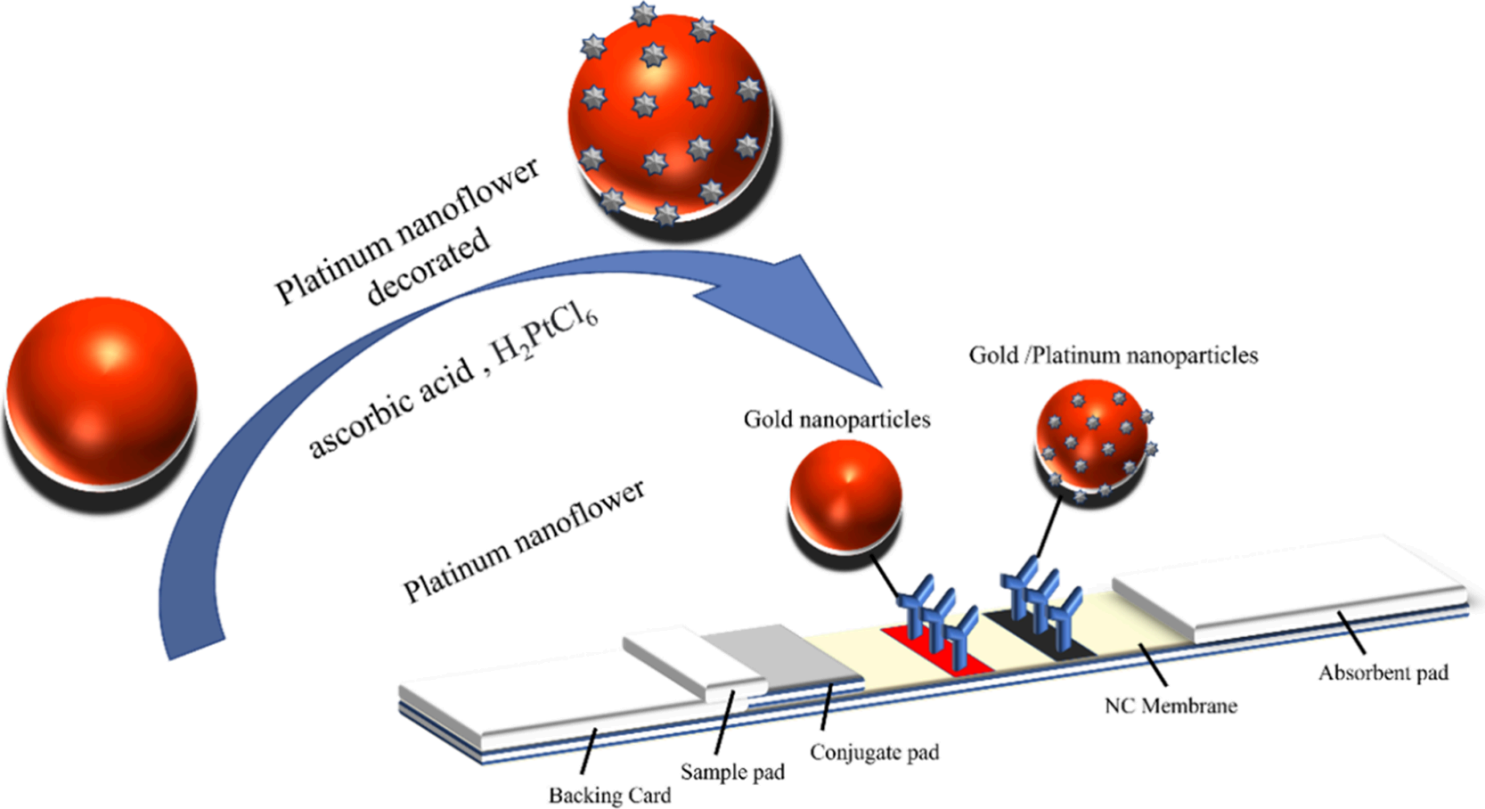
Schematic of the application of gold–platinum nanoparticles in the rapid screening of *C. difficile*.

We adjusted the pH of the gold–platinum nanoparticles (G3Pt, G4Pt, and G5Pt) to pH 9.5 by using potassium carbonate. Subsequently, Toxin B antibodies were added at a ratio of 1.6 μg/μL. We used a sample rotator to thoroughly mix the solutions for 1 h. After the addition of electrostatically adsorbed nanoparticles, a blocking buffer was introduced and stirring was continued for an additional 15 min. We centrifuged the solution at 10,000 rpm for 15 min and maintained a temperature of 4 °C. After removing the supernatant, we added a washing buffer. This process was repeated twice to complete the grafting of gold–platinum nanoparticles (Figure 1).

## Results and Discussion

### Basic Optical Properties of Gold Nanoparticles

**Figure 2:**
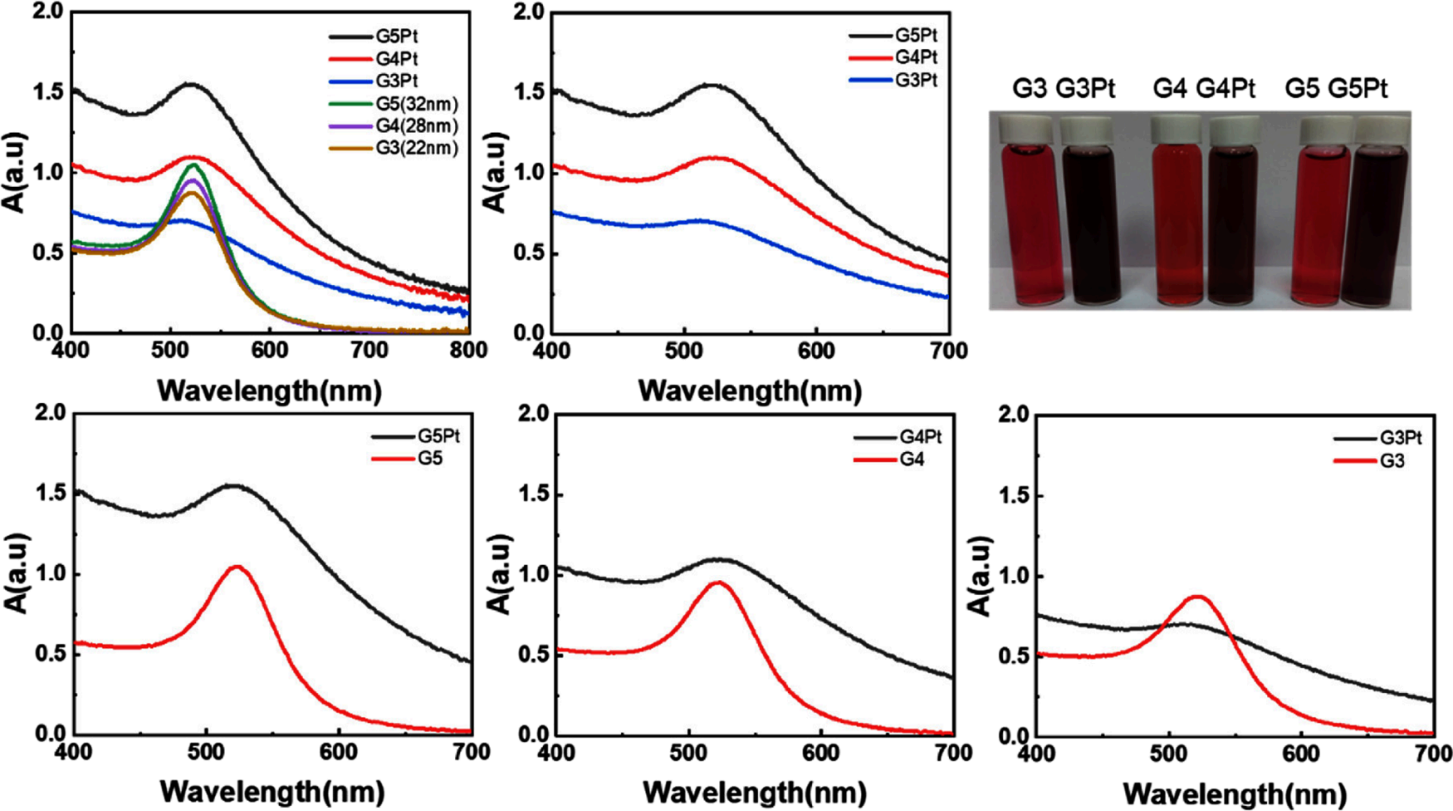
Optical properties of ultraviolet-visible spectroscopy analysis and naked-eye observation.

Ultraviolet-visible spectroscopy (Figure 2b) revealed that the absorption peaks of the gold nanoparticles shifted toward longer wavelengths as the particle size increased; the peaks observed for G3, G4, and G5 were at 22, 28, and 34 nm, respectively. The gold nanoparticles appeared red, whereas those modified with platinum exhibited a bluish-brown hue (Figure 2c). Moreover, the gold–platinum nanoparticles had a broader absorption range when compared with the pure gold nanoparticles, which absorbed primarily within the 500–600 nm range (Figure 2d–f).^22,23^ The spectral profiles of the gold–platinum nanoparticles indicated that they absorbed light across different wavelengths, resulting in colors distinct from the red of the gold nanoparticles. The results in Figure 2c were noted to be consistent with observations made for physical samples and with the ultraviolet-visible spectra (Figure 2d–f).

### Transmission Electron Microscopy Analysis

**Figure 3:**
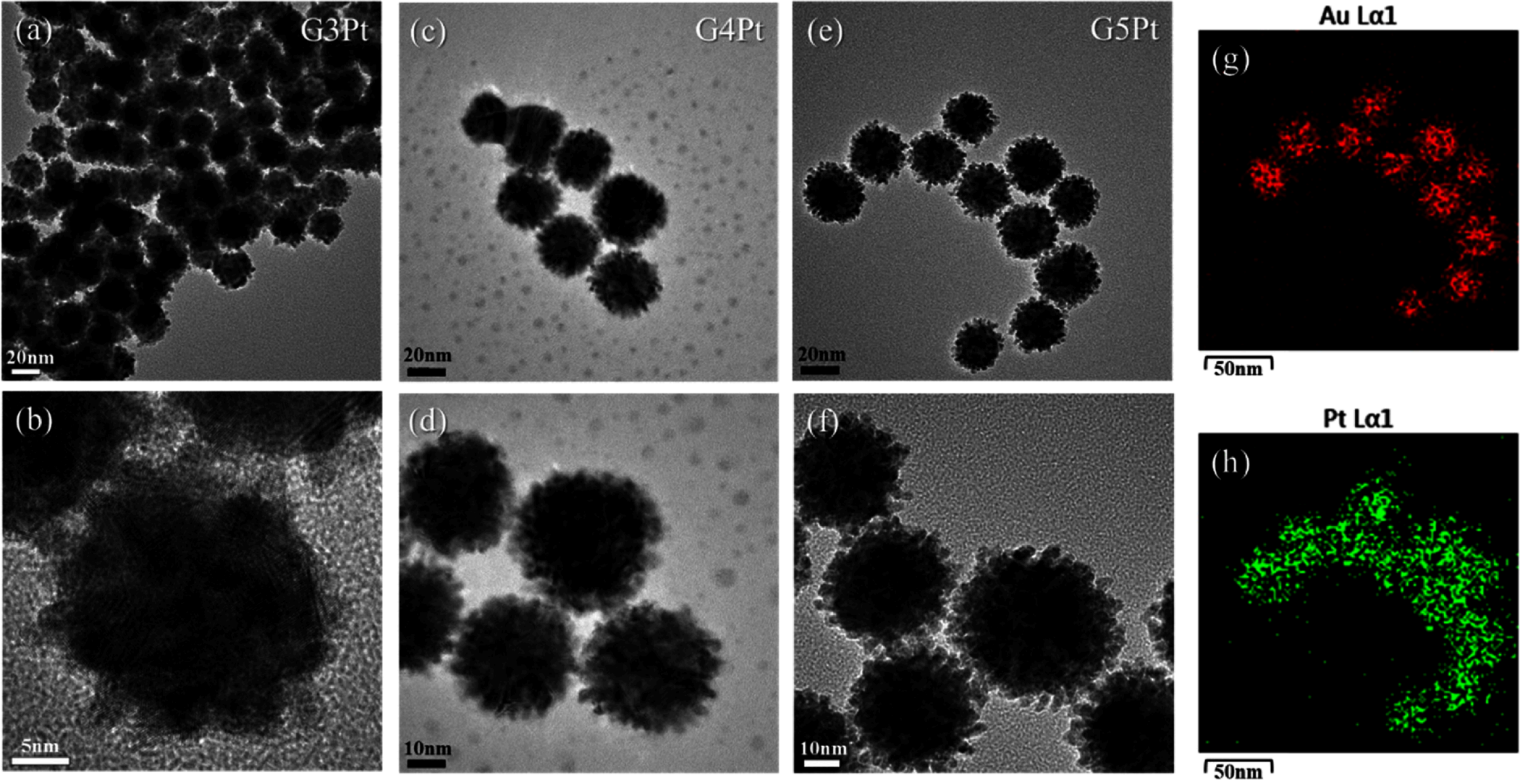
Transmission electron microscopy images of gold–platinum nanoparticles: (a, b) G3Pt nanoparticles at different magnifications, (c, d) G4Pt nanoparticles at different magnifications, (e, f) G5Pt nanoparticles at different magnifications, (g) elemental distribution of gold in G5Pt, and (h) elemental distribution of platinum in G5Pt.

Transmission electron microscopy was employed to analyze the surface morphology of the gold–platinum nanoparticles. The results (Figure 3) revealed composite nanoparticles of different sizes, with the G5Pt, G4Pt, and G3Pt nanoparticles having sizes of 39.1, 28.3, and 25.5 nm, respectively. In addition, we also analyzed the crystal plane information on Au/Pt nanoparticles (Figure S1). The surfaces of the nanoparticles were covered with point-like particles.^24,25^ Unlike spherical gold nanoparticles, the gold–platinum nanoparticles exhibited a larger surface area and differed in luster. Energy-dispersive X-ray spectroscopy (EDS) mapping was conducted to determine the elemental signal distribution of the prepared samples. The results indicated that the signals of gold exhibited a dense point distribution (Figure 3g), whereas the signals of platinum were observed around the gold nanoparticles (Figure 3h). The nanoparticles were confirmed to have a core/shell structure comprising gold and platinum (Figure 3g,h).

**Figure 4:**
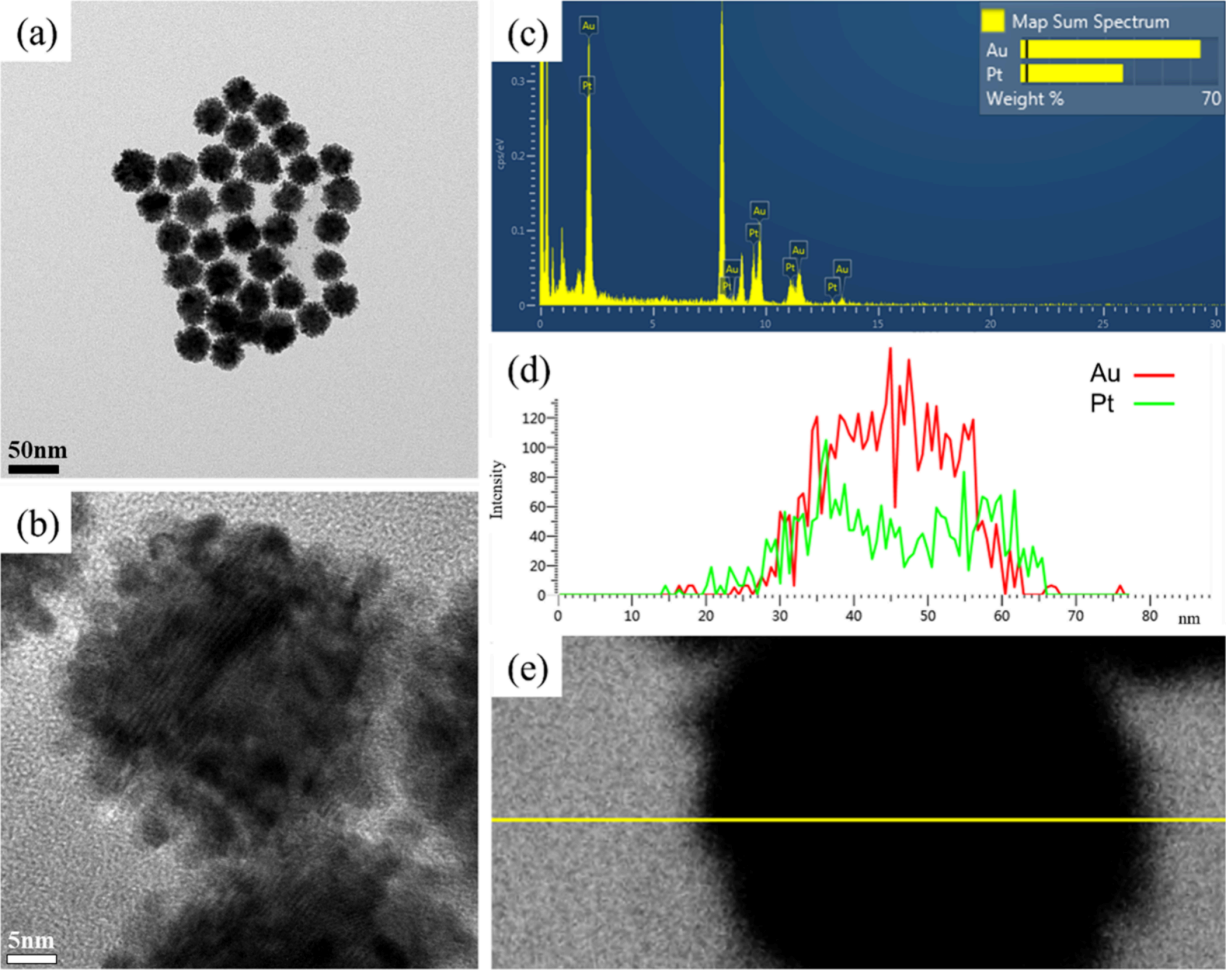
Transmission electron microscopy images of G4Pt nanoparticles. (a, b) TEM images at different magnifications, (c) energy scattering spectra of individual elements in G4Pt nanoparticles, and (d, e) penetration spectra and images of individual elements in G4Pt nanoparticles analyzed in line-scan mode under a conventional electron microscope.

Transmission electron microscopy was conducted in the line-scan mode to analyze the distribution of gold and platinum elements in the G4Pt nanoparticles (Figure 4). Analysis of the gold and platinum signals revealed that gold exhibited a relatively strong signal intensity, with its signals spanning from 30 to 60 nm (Figure 4d). By contrast, platinum had a weaker signal intensity, with its signals spanning from 25 to 65 nm. These findings indicate that the G4Pt nanoparticles exhibited a core/shell structure, with the gold nanoparticles encapsulated by a platinum shell. These results were noted to be consistent with the EDS mapping results, which revealed that the gold signals exhibited a dense point distribution (Figure 3g) and that the platinum signals were distributed around the gold nanoparticles (Figure 3h). In addition, the EDS results show that the atomic ratio is consistent with the concentration of our design ratio (Table S1). Accordingly, G4Pt nanoparticles can be confirmed to have a core/shell configuration (Figure 3g,h).

### DLS/Zeta Potential Analysis

**Figure 5:**
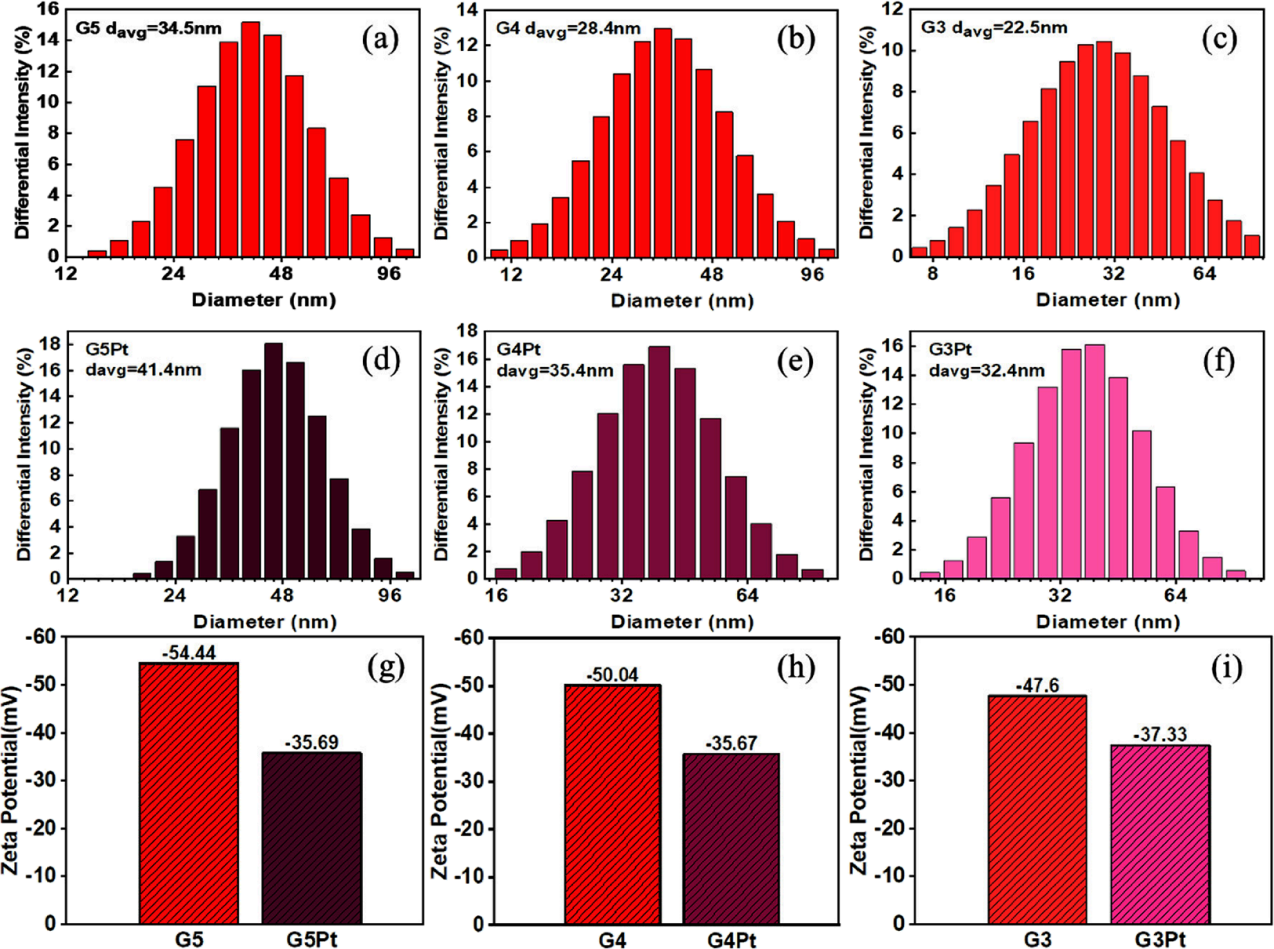
Average particle size and dispersion histograms for (a) G5, (b) G4, (c) G3, (d) G5Pt, (e) G4Pt, and (f) G3Pt nanoparticles. Zeta potential comparison between (g) G5 and G5Pt, (h) G4 and G4Pt, and (i) G3 and G3Pt nanoparticles.

Dynamic light scattering was used to analyze the synthesized gold nanoparticles in order to determine their dispersion characteristics and average particle sizes. The results revealed that the average sizes of the G5, G4, and G3 nanoparticles (which were not coated with platinum) were 34.5, 28.4, and 22.5 nm, respectively (Figure 5a–c). The average sizes of the G5Pt, G4Pt, and G3Pt nanoparticles (which were coated with platinum) increased by approximately 5–10 nm (Figure 5d–f). These results were noted to be consistent with the transmission electron microscopy results (Figure 3b–f), with the difference being approximately 2–7 nm. In addition, a more detailed comparison of TEM and DLS particle size analysis can be found in the Supporting Information (Table S2).

### LFAs

#### Protein A

**Figure 6:**
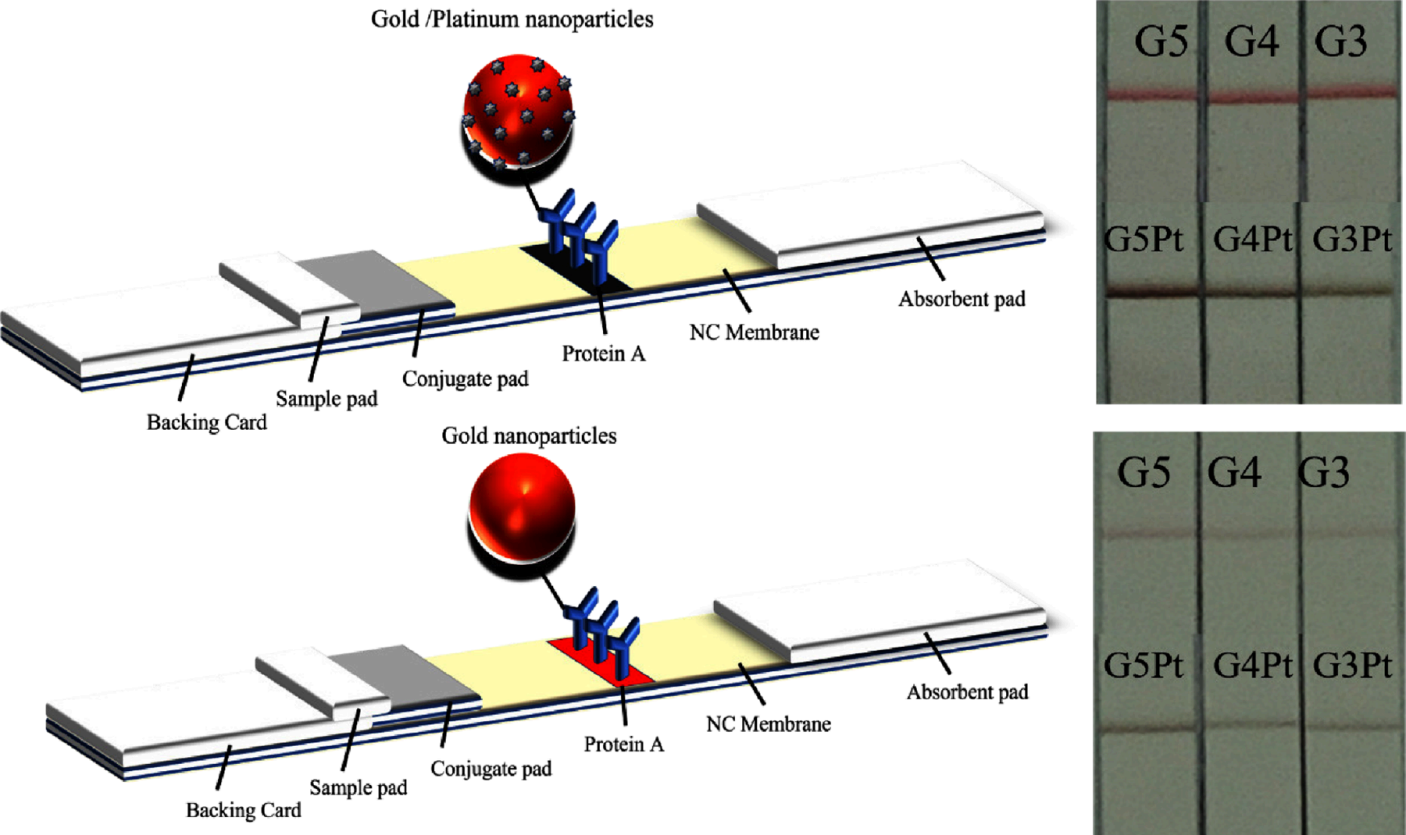
Experimental results of LFAs of gold nanoparticles and gold–platinum nanoparticles on Protein A.

We subjected the synthesized gold–platinum nanoparticles to an LSA. First, we electrostatically adsorbed the synthesized gold–platinum nanoparticles onto Protein A (Figure 6). Subsequently, we mixed the adsorbed colloidal gold solution with a configured running buffer. The mixture was then diluted 30 and 200 times. Next, we extracted 20 μL of the diluted solution for the LFA. After a 10 min assay, we noted that the nanoparticles in the mixture that was diluted 30 times exhibited minimal color changes, indicating a difference in expressiveness between traditional gold nanoparticles and gold–platinum nanoparticles. In the mixture that was diluted 200 times, the color of the G5 nanoparticles differed significantly from that of the G5Pt nanoparticles. This indicates that the G5Pt nanoparticles had superior color development abilities compared with that of the uncoated gold nanoparticles. Consequently, we selected G5Pt for further testing to evaluate its efficacy in the rapid screening of *C. difficile*.

#### Toxin B

**Figure 7:**
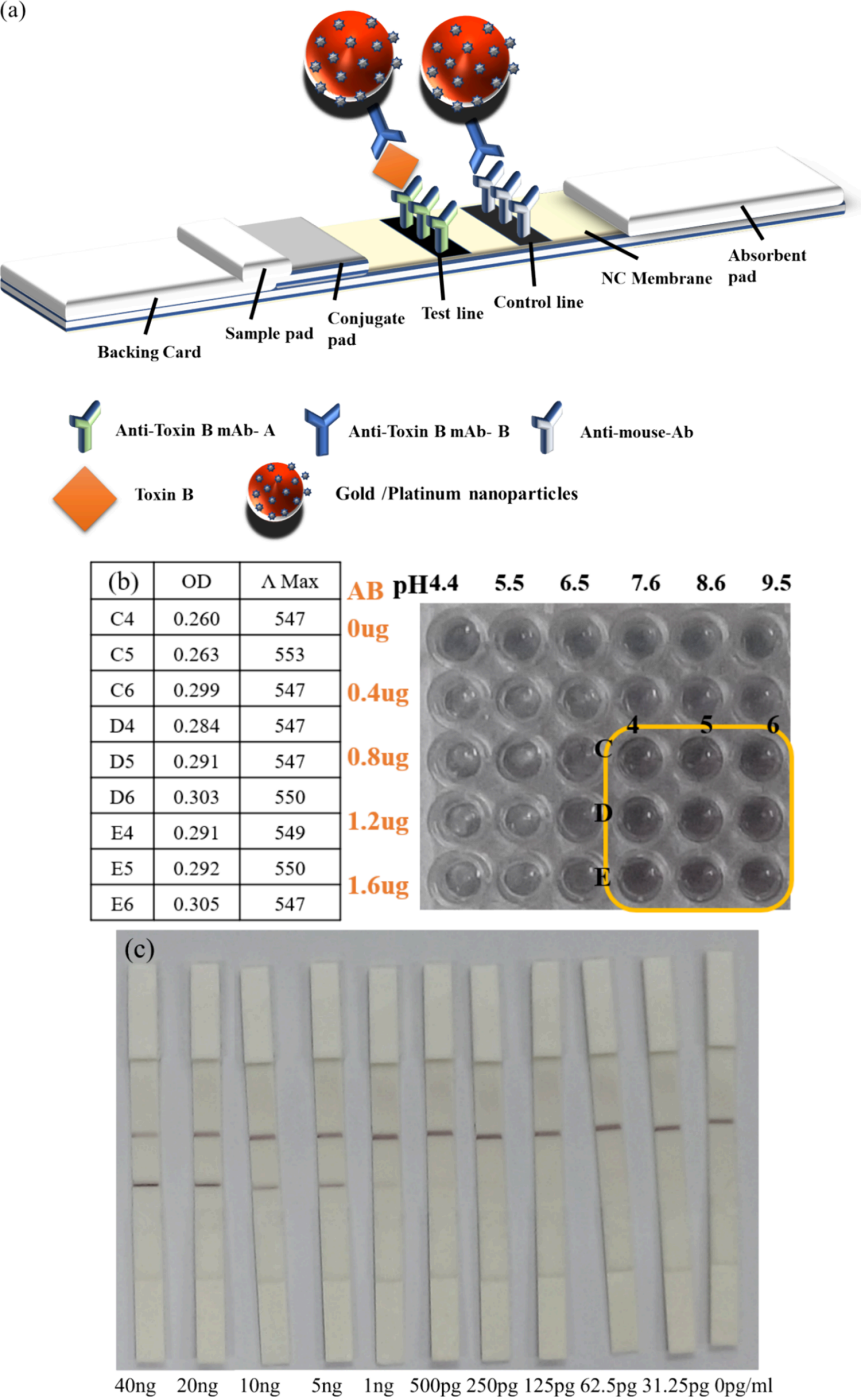
(a) Schematic of LFA for *C. difficile* screening. (b) Colorimetric detection results for G5Pt nanoparticles conjugated with *C. difficile* Toxin B under different pH conditions. (c) LFA results for G5Pt nanoparticles.

Figure 7b illustrates the colorimetric detection results for G5Pt nanoparticles conjugated with *C. difficile* Toxin B under various pH and Toxin B antibody protein concentration conditions;^26^ these conditions were tested to determine the optimal condition for the conjugation of the nanoparticles with the bacterium. The results indicated that a pH of 7.6–9.5 and antibody concentrations of 0.8–1.6 μg/100 μL constituted the most suitable conditions for conjugation. A pH of 9.5 and an antibody concentration of 1.6 μg/100 μL were used for subsequent detection of *C. difficile* Toxin B, as indicated by the highlighted wells in Figure 7b, and Figure 7c depicts the LFA results under this combination. After a 10 min LFA, the results revealed that the test line (T line) exhibited strong color development, with almost all nanoparticles reacting. However, when the virus concentration was 40 ng/mL, the control line (C line) appeared less distinct due to an overpowering reaction at the T line. As the concentration decreased, the color intensity of the C line began to recover. The line obtained using concentrations of 40 and 20 ng/mL exhibited deep black coloration, which transitioned to purple–brown at concentrations from 10 to 1 ng, with the lowest detection limit reaching 1 ng/mL. In addition, we also calculated the utilization value of Pt in the Au/Pt nanoparticles (Figure S2), which further indicates the broad applicability and future development potential of this synthesized Au/Pt nanoparticle method.

## Conclusions

Core/shell nanomaterials offer enhanced properties, including stability, conjugation, cytotoxicity reduction, and dispersibility, compared to simple nanomaterials, particularly in biological applications. In this study, we successfully synthesized gold–platinum nanoparticles by modifying the surface of gold nanoparticles with platinum nanoflowers. In comparison to pristine gold, advantages of Au/Pt presentation of data are (i) the increased surface-to-volume ratio renders higher antibody loading. (ii) Due to the broad band absorbance of platinum, the absorption peak of Au/Pt nanoparticles exhibits the combination result of the Au and Pt absorption spectrum, and the optical scattering depth is increased by decoration Pt nanoparticles. (iii) The larger particle size also contributes to the dark color and further intensifies it. Consequently, these gold–platinum nanoparticles not only exhibited a unique colorimetric response but also surpassed traditional gold nanoparticles in color development, particularly at low concentrations, when used in the detection of Protein A. Moreover, these nanoparticles demonstrated a chromogenic response to Toxin B in *C. difficile* at a concentration as low as 1 ng/mL, underscoring their considerable potential for application in LFAs.

## Supporting Information Available

The crystal planes of Au/Pt nanoparticles, TEM-EDS analysis of G4Pt nanoparticles, the comparison of size determined from TEM and DLS, and the comparison of platinum utilization values (PDF)
