# Supplementary material for: Study of Highly Efficient Au/Pt Nanoparticles for Rapid Screening of Clostridium difficile
Source: ACS Omega. 2024 May 24;9(23):24593–600. doi: 10.1021/acsomega.4c00722 (PMC11170621; doi:10.1021/acsomega.4c00722)
Supplement: Supplementary file 1 — ao4c00722_si_001.pdf [file ao4c00722_si_001.pdf]

Supporting Information

The Study of Highly Efficient Au/Pt Nanoparticles for Rapid Screening of Clostridium Difficile

Ying-Tsang Lu[1, 3†], Yu-Xlang Zeng[2†], Wu-Xiong Tsai[2], Hsin-Chang Huang[2, 4], Ming-Yuan Tsai[4], Yong Diao[1], Wei-Hsuan Hung[2*]*

1 School of Medicine, Huaqiao University,

No.269 Chenghua North Rd. Quanzhou, Fujian, China 362021

2 Institute of Material Science and Engineering, National Central University,

No. 300, Zhong-da Rd., Zhongli District., Taoyuan City 320, Taiwan, R.O.C.

3 STRONG BIOTECH CORPORATION,

7f., No. 32, Sec. 1, Chenggong Rd., Nangang Dist., Taipei City 11570, Taiwan, R.O.C.

4 TRIPOD NANO TECHNOLOGY CORPORATION

No. 3, Gongye 12th Rd., Pingzhen Dist., Taoyuan City 324403, Taiwan, R.O.C.

†The authors contributed equally to this work.

* Corresponding Authors: E-mail: diaoyong@hqu.edu.cn(Yong Diao); hungwh@ncu.edu.tw(W. H. Hung)

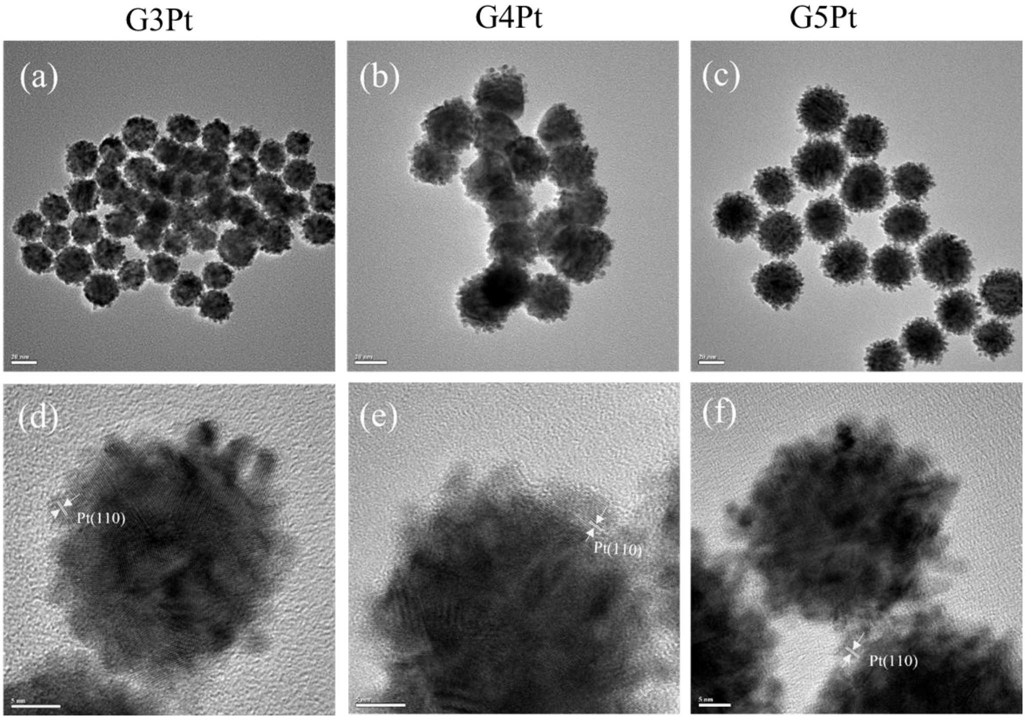

Figure S1. The TEM image of Au/Pt nanoparticles.

Table S1. TEM-EDS analysis of G4Pt nanoparticles.

Element	Line Type	k Factor	k Factor type	Absorption Correction	Wt%	Wt% Sigma	Atomic %
Pt	L series	2.630	Theoretical	1.00	35.01	0.76	35.23
Au	L series	2.672	Theoretical	1.00	64.99	0.76	64.77
Total:					100.00		100.00

Table S2. The size of Au/Pt nanoparticles measured using TEM and DLS respectively.

Instrument	G3Pt size(nm)	G4Pt size(nm)	G5Pt size(nm)
TEM	25.5	28.3	39.1
DLS	32.4	35.4	41.4

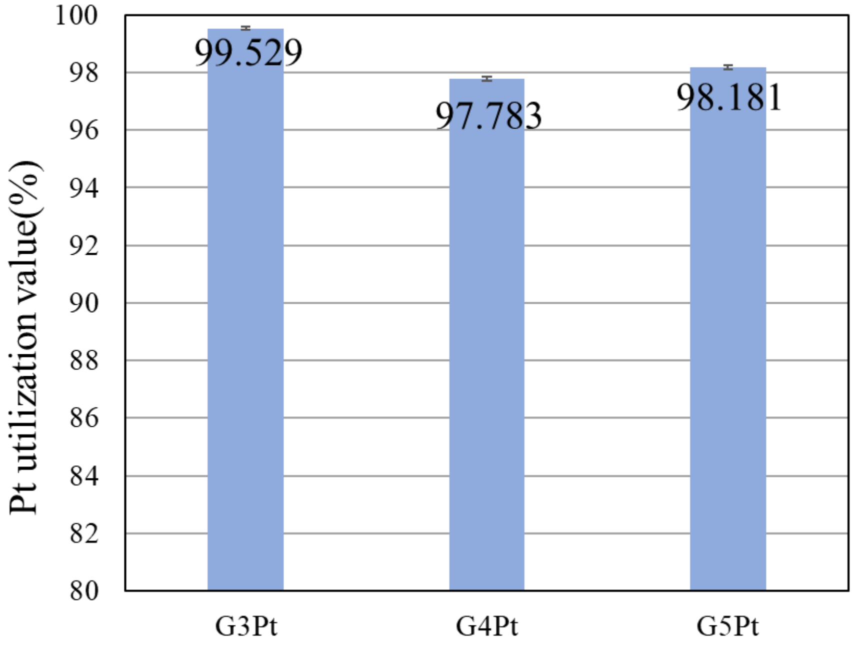

Figure S2. The comparison of platinum utilization value of G3Pt/G4Pt/G5Pt.
